# Inhibition-excitation balance in the parietal cortex modulates volitional control for auditory and visual multistability

**DOI:** 10.1038/s41598-018-32892-3

**Published:** 2018-09-28

**Authors:** Hirohito M. Kondo, Daniel Pressnitzer, Yasuhiro Shimada, Takanori Kochiyama, Makio Kashino

**Affiliations:** 1 School of Psychology, Chukyo University, Nagoya, Aichi Japan; 2 Human Information Science Laboratory, NTT Communication Science Laboratories, NTT Corporation, Atsugi, Kanagawa Japan; 3 Laboratoire des Systèmes Perceptifs, CNRS UMR 8248, Paris, France; 4 Département d’Études Cognitive, École Normale Supérieure, Paris, France; 5 Brain Activity Imaging Center, ATR-Promotions, Seika-cho, Kyoto Japan; 6 Department of Cognitive Neuroscience, Advanced Telecommunications Research Institute International, Seika-cho, Kyoto Japan; 7 Sports Brain Science Project, NTT Communication Science Laboratories, NTT Corporation, Atsugi, Kanagawa Japan; 8 School of Engineering, Tokyo Institute of Technology, Yokohama, Kanagawa Japan

## Abstract

Perceptual organisation must select one interpretation from several alternatives to guide behaviour. Computational models suggest that this could be achieved through an interplay between inhibition and excitation across competing types of neural population coding for each interpretation. Here, to test for such models, we used magnetic resonance spectroscopy to measure non-invasively the concentrations of inhibitory γ-aminobutyric acid (GABA) and excitatory glutamate-glutamine (Glx) in several brain regions. Human participants first performed auditory and visual multistability tasks that produced spontaneous switching between percepts. Then, we observed that longer percept durations during behaviour were associated with higher GABA/Glx ratios in the sensory area coding for each modality. When participants were asked to voluntarily modulate their perception, a common factor across modalities emerged: the GABA/Glx ratio in the posterior parietal cortex tended to be positively correlated with the amount of effective volitional control. Our results provide direct evidence implicating that the balance between neural inhibition and excitation within sensory regions resolves perceptual competition. This powerful computational principle appears to be leveraged by both audition and vision, implemented independently across modalities, but modulated by an integrated control process.

## Introduction

Perceptual multistability describes an intriguing situation, whereby an observer reports random changes in conscious perception for a physically unchanging stimulus^1,2^. Multistability is a powerful tool with which to probe perceptual organisation, as it highlights perhaps the most fundamental issue faced by perception for any reasonably complex natural scene. And because the information encoded by sensory receptors is never sufficient to fully specify the state of the outside world^3^, at each instant perception must always choose between a number of competing alternatives. In realistic situations, the process produces a stable and useful representation of the world. In situations with intrinsically ambiguous information, the same process is revealed as multistable perception.

A number of theoretical models have converged to pinpoint the generic computational principles likely to be required to explain multistability, and hence perceptual organisation^4–9^. All of these models consider three core ingredients: inhibition between competing neural populations, adaptation within these populations, and neuronal noise. The precise role of each ingredient and their respective importance is still being debated. Noise is introduced to induce fluctuations in each population and initiate the stochastic perceptual switching in some models^7–9^, whereas switching dynamics are solely determined by inhibition in others^5,6^. Functional brain imaging in humans has provided results qualitatively compatible with those computational principles at several levels of the visual processing hierarchy^10^. But, for most functional imaging techniques in humans such as fMRI or MEG/EEG, changes in activation are difficult to trace back to neural mechanisms. For instance, an increase in activation in a given brain region could be the result of more excitation from upstream parts of the recruited network, or, equivalently, of less local inhibition. In addition, such an increase in activation could be associated with either more excitatory or more inhibitory projections to downstream parts of the network^11^. Therefore, direct experimental evidence of the implication of inhibition, excitation, or both, as theorized by the models, has been difficult to obtain.

**Figure 1:**
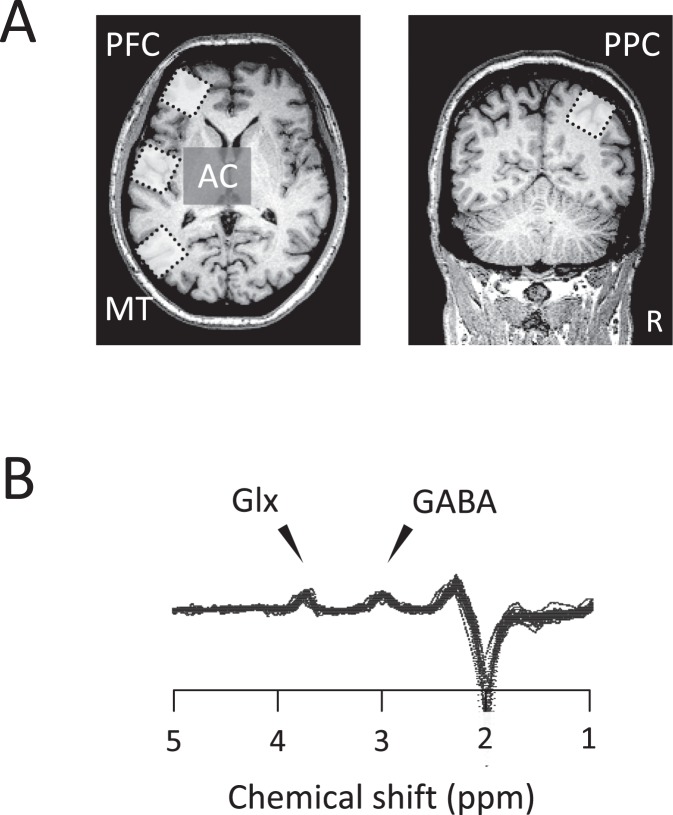
The size and location of voxels and the edited GABA spectra (*N* = 36). (**A**) The voxel in the auditory cortex (AC) included the Heschl gyrus and the anterior part of the temporal plane. The voxel in the motion-sensitive area (MT) was placed at the ventrolateral occipital cortex. The voxel in the prefrontal cortex (PFC) was located at the anterior part of the middle frontal gyrus. The voxel in the posterior parietal cortex (PPC) was centred on the intraparietal sulcus. (**B**) The GABA and Glx peaks were calculated using the differences between AC spectra obtained by editing radio frequency on/off pulses. GABA, γ-aminobutyric acid; Glx, glutamate-glutamine; R, right.

We used magnetic resonance spectroscopy (MRS) to obtain non-invasive, *in vivo* measurements of γ-aminobutyric acid (GABA) and glutamate-glutamine (Glx) concentrations in the human brain^12^ (Fig. 1). Using the same MRS technique, van Loon *et al*. (2013) investigated the link between GABA and Glx concentrations and visual multistability tasks for three different types of stimuli: structure from motion, binocular rivalry, and motion-induced blindness^13^. They showed that in all cases a higher GABA concentration was associated with longer percept durations. Intriguingly, a link with Glx was only found in the stimulus of structure from motion, so the inhibition-excitation balance model was not fully supported by their results. A more recent study using similar auditory streaming has demonstrated that the Glx concentration in the auditory cortex (AC) was related to the dominance of “segregated” percepts, where a complex scene is broken into separate streams^14^. In another study investigating the priming of ambiguous motion by non-ambiguous motion, Glx concentration in the prefrontal cortex (PFC) was correlated with the dominance of visual motion assimilation between context and test^15^. These two later sets of results point to the usefulness of studying the neurotransmitter concentration with MRS in relation to multistable perception tasks, but this is not tested directly in the inhibition-excitation balance model. Thus, even if there are now results strongly suggesting that neural inhibition or excitation is an integral part of perceptual selection, the implication of a more specific model of the inhibition-excitation balance remains to be demonstrated.

**Figure 2:**
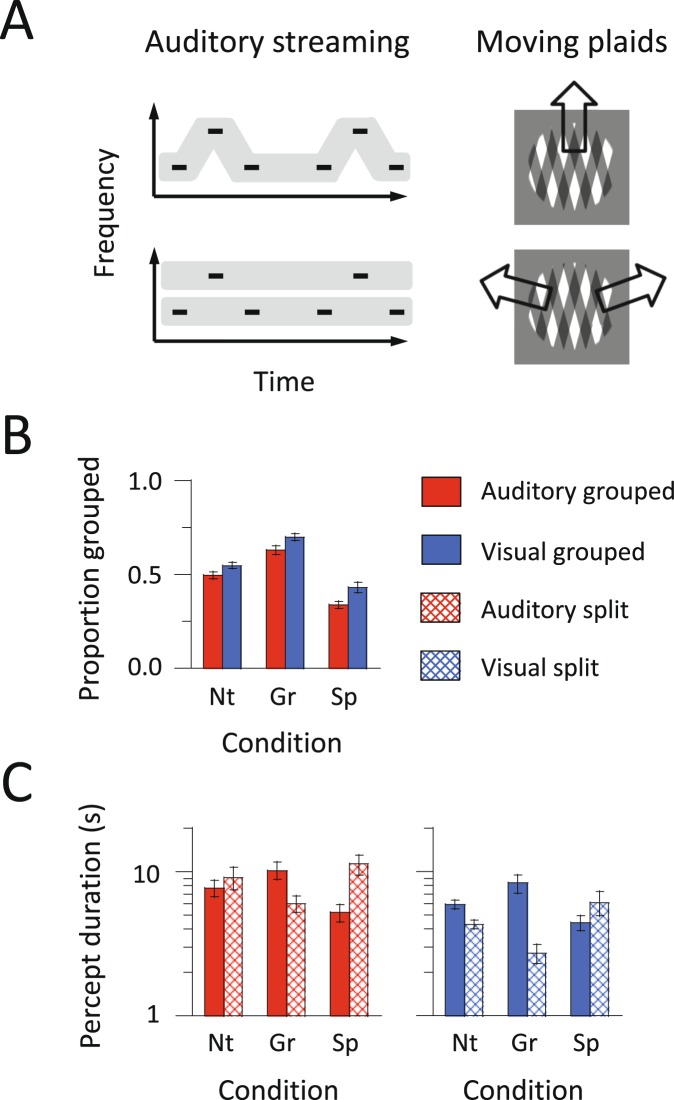
Stimuli and behavioural results for auditory and visual multistability. (**A**) Schematic representation of stimuli. A sound sequence of triplet tones was presented in auditory streaming. The auditory stimulus produces perceptual switches between one and two streams. Two rectangular gratings were moving in plaids. The visual stimulus induces perceptual switches between upward grouped and sideward split motion. (**B**,**C**) The average proportion of time spent in grouped percepts is shown as a function of condition (Neutral, Grouped, and Split) and input modality (auditory and visual presentation). Red and blue bars indicate the results for auditory streaming and moving plaids, respectively. The duration of alternating percepts is converted to a logarithmic scale for display purposes. Error bars represent the standard error of means.

Here, we examined the interplay between inhibition and excitation for multistable stimuli, comparing two sensory modalities using auditory streaming^16,17^ and plaid motion^18,19^ (Fig. 2A). The use of two modalities has several advantages. First, given the spatial resolution of MRS, it is possible to image brain regions involved in the processing of auditory or visual stimuli separately. Auditory streaming has neural correlates in AC^17,20^, whereas moving plaids induce activity in the motion-sensitive area (MT) of the visual cortex^21,22^. On the basis of previous findings and in relation to the theoretical model, we hypothesized that higher GABA/Glx ratios (more inhibition) in AC and MT might slow alternations and thus lead to longer percept durations in auditory streaming and moving plaids. Second, in spite of their superficial differences, auditory streaming and moving plaids both involve a basic competition between a one-object (Grouped) and a two-object (Split) interpretation. Perhaps as a consequence, deep similarities in the behavioural characteristics of multistability have been found for these stimuli^16,23^. Comparing the two modalities is thus a useful test of the generic principles of perceptual organisation, beyond specific stimulus characteristics. Finally, the comparison across modalities allows to address one of the longest-standing questions regarding multistability^10,24,25^, namely is the multistable selection process distributed throughout the brain, including in some low-level sensory areas, or is there a dominant role for higher-level regions through cognitive control? In particular, a causal role for the posterior parietal cortex (PPC) and PFC has long been argued as regards perceptual switching in auditory and visual multistability^26–29^, but this has recently been disputed^30^. Accordingly, we tested the further hypothesis that the GABA/Glx ratios in non-sensory brain regions, here PPC and PFC, could be involved in the behavioural characteristics of auditory and visual multistability. The PPC in particular has not been examined in previous studies using MRS.

The role of higher brain regions could be to initiate the switches, but it could also be to modulate perception when the participant tries to exert some control over it. To test for this last hypothesis, we manipulated the volition of participants during the task (also sometimes referred to as selective attention or willful intent) in addition to collecting spontaneous reports. It is known that participants are to some extent able to influence their percepts in multistable situations, either when asked to slow down or speed up the rate of reversals^31–34^ or when asked to favour one of the two possible interpretations^16,35^. Interestingly, if a top-down volitional mechanism originates from PPC or PFC, it should reasonably be expected to be shared across perceptual modalities and thus correlate with behaviour irrespective of the modality of entry.

In brief, we acquired spectroscopy measurements in four brain regions. This provided one estimate of neurotransmitter balance per region, per participant. We then explored the correlations, over participants, between these measures of inhibition-excitation balance and inter-individual variations in perception - as measured in a separate behavioural session.

## Results

### Behavioural percept durations

We first investigated the pattern of reported percepts under different volitional conditions, namely Neutral for spontaneous reports and Grouped or Split for the two different instruction cues (Fig. 2B). A repeated-measures analysis of variance (ANOVA) showed that the proportion (mean ± standard error) of reported grouped percepts was larger overall for moving plaids (55.9 ± 1.2%) than for auditory streaming (48.8 ± 1.3%): *F*_(1, 35)_ = 22.98, *η*_p_^2^ = 0.40, *p* < 0.001. For both modalities, as expected, the proportion of grouped percepts increased under the Grouped condition (66.4 ± 1.8%) compared with the Neutral condition (52.3 ± 1.4%). Also, as expected, the proportion of grouped percepts decreased under the Split condition (38.4 ± 2.1%): *F*_(2, 70)_ = 60.67, *η*_p_^2^ = 0.63, *p* < 0.001. The interaction between modality and condition was not significant: *F*_(2, 70)_ = 0.76, *η*_p_^2^ = 0.02, *p* > 0.47. This lack of interaction indicates that the amount of volitional control did not differ between auditory streaming and moving plaids.

We then characterized the finer effects of volitional intent on percept durations (Fig. 2C). For auditory streaming, a 2 × 3 ANOVA revealed an interaction between percept type and volitional condition: *F*_(2, 70)_ = 33.03, *η*_p_^2^ = 0.49, *p* < 0.001. The duration of grouped percepts according to the volitional condition displayed the following pattern: Grouped (10.2 ± 1.4 s) > Neutral (7.7 ± 1.0 s) > Split (5.2 ± 0.7 s). For split percepts, the pattern was reversed: Split (11.3 ± 1.8 s) > Neutral (9.1 ± 1.6 s) > Grouped (6.0 ± 0.8 s). Similar results were observed for moving plaids, with an interaction between percept type and condition: *F*_(2, 70)_ = 46.44, *η*_p_^2^ = 0.57, *p* < 0.001. The duration of grouped percepts according to the volitional condition displayed the following pattern: Grouped (8.3 ± 1.2 s) > Neutral (5.9 ± 0.4 s) > Split (4.4 ± 0.5 s). For split percepts, the pattern was reversed: Split (6.1 ± 1.2 s) > Neutral (4.3 ± 0.3 s) > Grouped (2.7 ± 0.4 s).

In summary, we observed the same effect of volitional control for both modalities, with volition resulting in increased durations for the target percepts and shortened durations for the non-target percepts.

### Behavioural correlations across modalities

**Figure 3:**
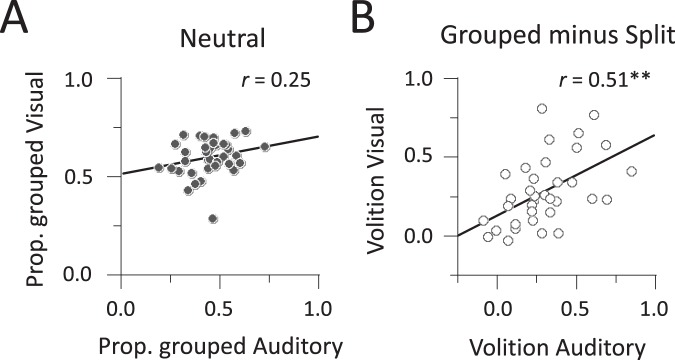
Relationship between perceptual multistability and volitional control. (**A**) Correlation of the proportion of grouped percepts between auditory streaming and moving plaids. Symbols indicate individual data under the Neutral conditions. (**B**) Correlation of the amount of effective volitional control between auditory streaming and moving plaids. The amount of effective volitional control is computed by subtracting the proportion of grouped percepts between the Grouped and Split conditions. **p < 0.01.

Next, we looked for correlations across modalities under the different conditions of volitional control. Under the Neutral condition, the correlation between the proportion of grouped percepts across modalities did not reach statistical significance: *r* = 0.25 (95% confidence interval, −0.08 to 0.53), *p* > 0.14 (Fig. 3A). This suggests a functional independence of modalities in the Neutral condition: participants who tended to perceive the grouped interpretation in audition were not the same as those who tended to perceive the grouped interpretation in vision.

We extended the analysis to situations where volitional control was applied. We computed the amount of effective volitional control by using the proportion of grouped percepts under the Grouped versus Split conditions. The amount of effective volitional control as a whole did not differ between modalities: auditory streaming (29.1 ± 3.6%) versus moving plaids (26.9 ± 3.5%), *t* = 0.59, *η*^2^ = 0.01, *p* > 0.50. However, on a participant-by-participant basis, the amount of effective volitional control was positively correlated across modalities: *r* = 0.51 (95% CI, 0.22 to 0.72), *p* < 0.001 (Fig. 3B). This means that in contrast to the Neutral case, individuals who were better at controlling their auditory percepts were also better at controlling their visual percepts.

In summary, we found no evidence for correlation across modalities in the Neutral condition. However, a correlation appeared under conditions of volitional control. This suggests an additional common factor across modalities that shapes perceptual alternations under conditions of volitional control only.

### GABA/Glx ratio compared with percept durations

**Figure 4:**
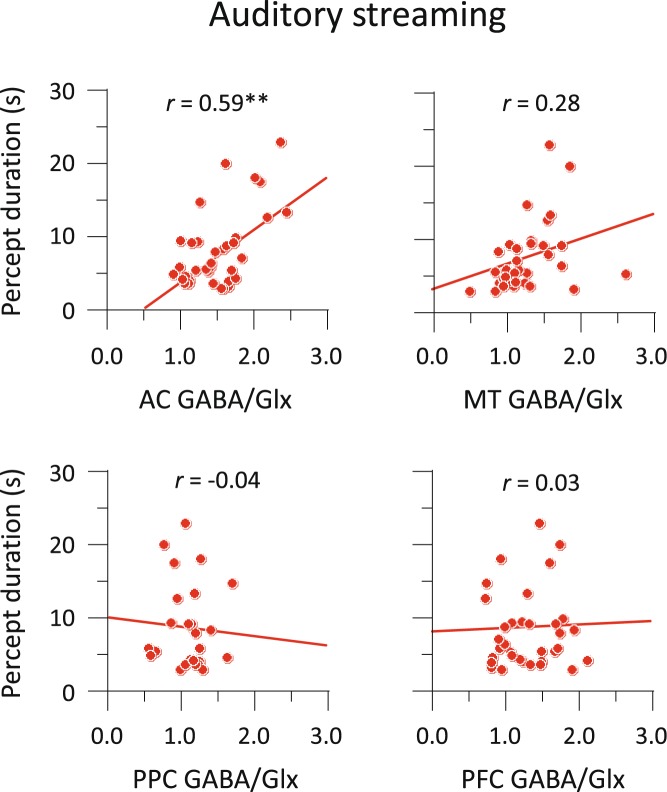
Correlations between MRS and behavioural results in auditory streaming. Scatter plots with linear regression fit for the relationship between GABA/Glx ratios and median percept durations. Circles indicate individual data under the Neutral conditions. **p < 0.01.

**Figure 5:**
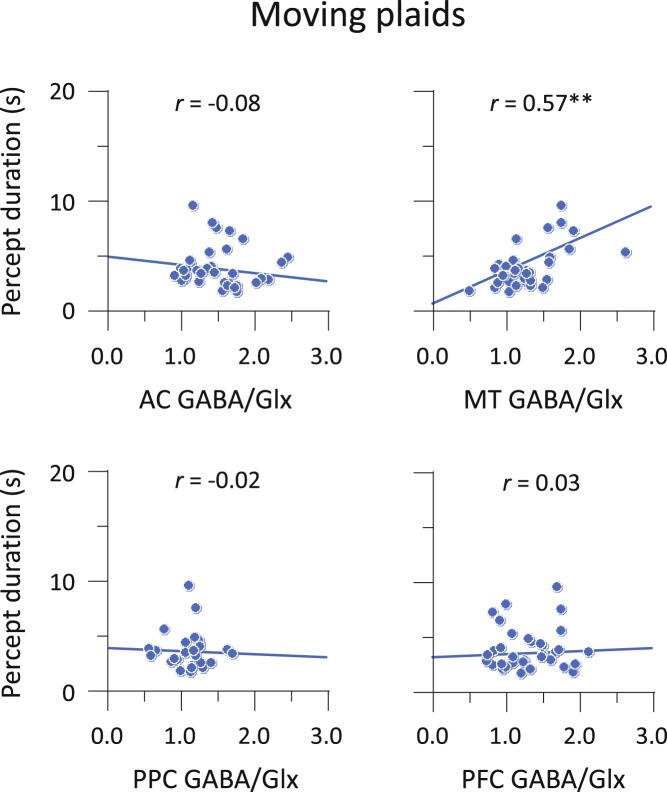
Correlations between MRS and behavioural results in moving plaids. Scatter plots with linear regression fit for the relationship between GABA/Glx ratios and median percept durations. Circles indicate individual data under the Neutral conditions. **p < 0.01.

Figure 4 shows individual results comparing the percept duration with the GABA/Glx ratio. Auditory percept durations were positively correlated with the GABA/Glx ratios in the AC voxel: *r* = 0.59 (95% CI, 0.32 to 0.77), *p* < 0.001. Importantly, the correlation in AC with auditory behaviour was larger than that in MT, PPC or PFC: *t* > 2.08, *p* < 0.05. Mirroring this observation, visual percept durations were positively correlated with the GABA/Glx ratios in the MT voxel: *r* = 0.57 (95% CI, 0.29 to 0.76), *p* < 0.001. The correlation in MT was larger than that in AC, PPC or PFC: *t* > 2.41, *p* < 0.05 (Fig. 5). We found a similar pattern of correlations in terms of GABA- and Glx-alone measures (Supplementary Table S1). In sensory areas, the correlations of the GABA/Glx ratios with percept durations were generally greater than those of the GABA and Glx concentrations, but the overall pattern of result was identical.

In summary, we observed strikingly similar patterns for the auditory and visual cases: the GABA/Glx ratio within the sensory area coding for the stimulus was reliably correlated with percept durations. In both modalities, participants who had higher GABA/Glx ratios displayed longer average percept durations. The GABA and Glx concentrations accounted for a sizeable proportion of the variance, which is remarkable given the many possible sources of experimental noise potentially involved in each measure. Finally, these correlations were, highly specific: they were only observed in the sensory area coding for the modality of interest, and not in other sensory areas nor prefrontal or parietal areas.

### GABA/Glx ratio compared with volitional control

We examined whether the GABA/Glx ratio could account for the common factor between audition and vision under conditions of volitional control suggested by the behavioural analysis. This was accomplished by correlating the amount of behavioural volitional control (Fig. 3B) with the GABA/Glx ratio in the AC, MT, PFC, and PPC voxels.

**Figure 6:**
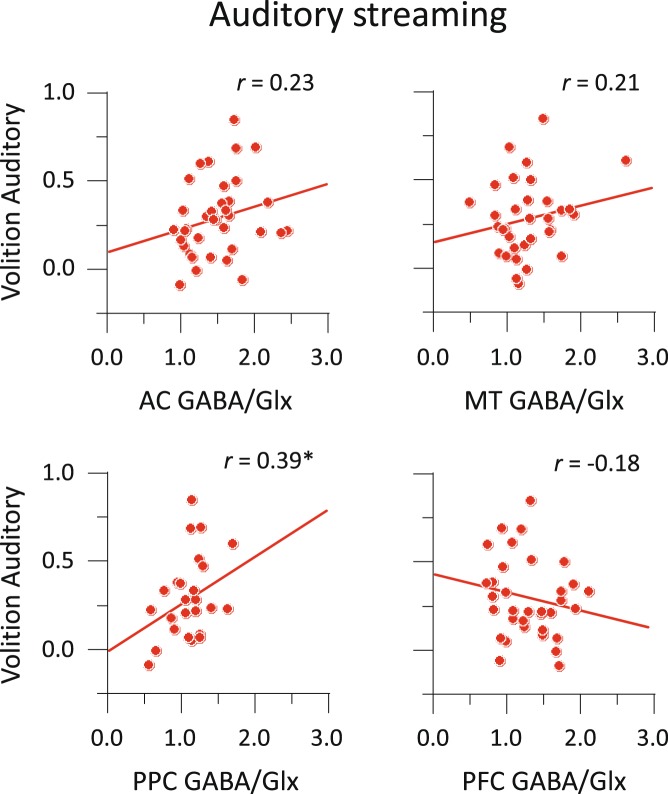
Correlations between MRS measure and volitional control for auditory streaming. Amounts of effective volitional control as a function of GABA/Glx ratio. *p < 0.05.

**Figure 7:**
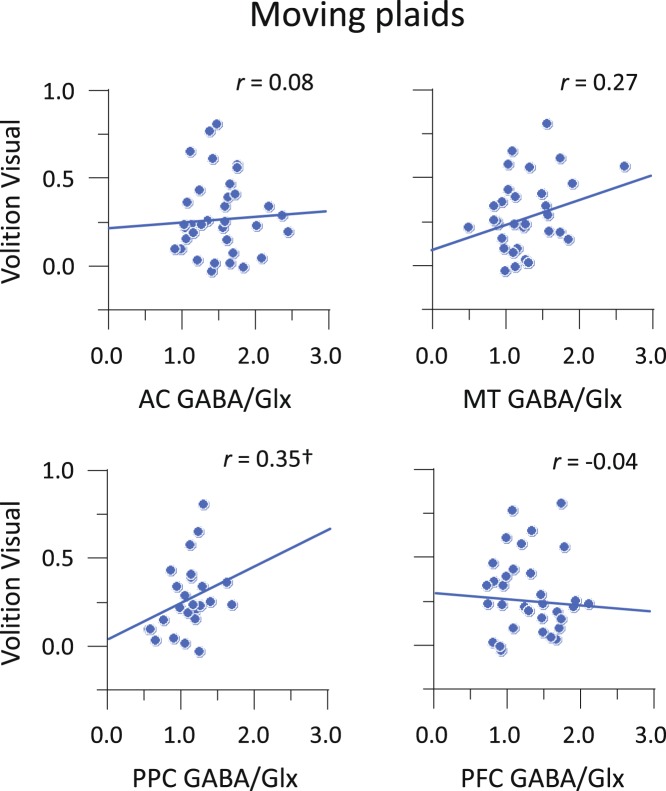
Correlations between MRS measure and volitional control for moving plaids. Amounts of effective volitional control as a function of GABA/Glx ratio. ^†^*p* < 0.10.

For auditory streaming, the GABA/Glx ratio in PPC showed a significant correlation with the amount of effective volitional control: *r* = 0.39 (95% CI, 0.00 to 0.67), *p* < 0.05 (Fig. 6). There was no trend towards correlation between the amount of effective volitional control and the GABA/Glx ratio in any of the other voxels: *r* < 0.23 in all cases. The correlation in PPC with volitional control was larger than that in PFC: *t* = 2.18, *p* < 0.05. For moving plaids, there was a marginal correlation between the amount of effective volitional control and the GABA/Glx ratio in PPC: *r* = 0.35 (95% CI, −0.04 to 0.64), *p* = 0.082, in the same direction as that observed for audition and the PPC voxel (Fig. 7). Again, we found no significant correlation between the amount of effective volitional control in vision and GABA/Glx ratio in any of the other voxels: *r* < 0.27. The correlation in PPC with volitional control was larger than that in PFC: *t* = 2.03, *p* < 0.05. Both sets of results were thus overall similar for audition and vision, especially considering that the difference between the auditory and visual correlation coefficients in PPC was not significant (*t* < 1, *p* > 0.80). When the same analyses of volitional control were repeated using GABA and Glx measures separately, correlations did not reach statistical significance (see Supplementary Table S1), suggesting that the ratio GABA/Glx is a more accurate measure of volitional control of perception.

Therefore, even if some of the analyses just fall short of significance, the results suggest that the best candidate to explain the correlation between auditory and visual volitional control is the inhibition-excitation balance in PPC, but not in PFC.

## Discussion

Using a comparison between perceptual multistability in audition and vision, we showed that higher GABA/Glx ratios within sensory regions correlated with longer percept durations. This is precisely predicted by computational models of perceptual organisation based on a balance of excitation and inhibition^13,25^. Importantly, the correlation was only found within sensory areas specialised for the stimulus modality: AC for audition and MT for vision. Under conditions of spontaneous reports, such a correlation did not extend to higher brain areas such as PPC and PFC. However, when volitional control was applied, a behavioural correlation across modality appeared and the GABA/Glx ratio in PPC tended to be linked to the effectiveness of volitional control.

### Direct evidence in humans of distributed competition during perceptual organisation

The first set of novel findings in the present study relates to the modality-specific nature of the correlation between GABA and Glx concentrations and behaviour, in the neutral-instructions case. On a methodological level, this specificity precludes an interpretation of the MRS results as a generic effect of GABA/Glx ratios in the brain, because for instance percept durations in the auditory task were not correlated with GABA/Glx ratios in MT. Rather, the modality-specific but qualitatively similar correlations can be interpreted as a common computational principle, namely the balance between inhibition and excitation, implemented independently across each sensory modality^36^.

The nature of the correlation (more inhibition leading to longer percepts) is consistent with previous MRS results obtained with some visual stimuli^13^, extending them to yet another visual motion stimulus. To the best of our knowledge, this is the first direct experimental evidence for such processes in the auditory modality. This novel finding has theoretical importance because the ‘features’ in competition for visual stimuli, such as the dominant percept in binocular rivalry, are better understood than those at play in auditory streaming, which presumably involve a strong timing element. The traditional cortical accounts of auditory streaming do not in fact require an inhibition-excitation mechanism^37,38^. Our data show that framing the streaming problem in terms of competing neural populations^39,40^ or the accumulation of evidence with an inhibitory process to effect classification^41^ may be promising avenues for guiding future neurophysiological investigations^25^.

### Idiosyncratic variability in multistable perception

Our results add another cause of the puzzling but well-documented idiosyncratic variability in the behavioural characteristics of perceptual multistability^42^. Genotype variations impacting the dopamine system have been found to correlate with individual differences in auditory streaming and verbal transformations^23^. Structural correlates have been uncovered for visual bistable dynamics, for cortical volume in frontal and visual areas^43^, for cortical volume in parietal regions^29^, as well as for inter-region connectivity^44^. Here, we demonstrate that the relative balance of the GABA and Glx concentrations is also an idiosyncratic factor affecting percept durations for both visual and auditory multistability. This has potential clinical implications, as developmental disorders that occur during the maturation of the GABAergic system can lead to the long-term disruption of cortical circuits^45^, with decreases in GABA production^46^. In addition, alterations in glutamate signalling in the cortex can induce sensory memory impairments^47^ and the recurrence of auditory hallucinations^48^. Thus, our findings have implications pertaining to the impact of disorders of the GABAergic and glutamatergic systems on mid-level functions such as perceptual organisation.

### Possible mechanisms of volitional control

Another set of findings relates to the effect of volition. The behavioural data showed that volition could both lengthen a target percept and shorten an unwanted percept. If we equate volitional intent with a strengthening of the target percept, this seems at odd with Levelt’s second proposal for binocular rivalry^49^ and with our own previous reports^16^. However, there are other such examples of the lengthening of the strengthened percept in vision that depend on details of the experimental situation^50^. It would seem that the larger cohort tested here allowed the measurement of such an effect for auditory streaming.

We demonstrated that a higher GABA/Glx ratio in PPC tended to be associated with a larger magnitude of effective volitional control for perceptual organisation. The correlation just failed to reach our statistical significance criterion, perhaps because the sample size for the PPC voxel analysis was smaller than for other voxels, or because the imaging and behavioural data were collected further apart in time than for other voxels. In any case, the correlation with the GABA/Glx ratio in PPC was stronger than in the PFC region tested here.

A recent study using an attentional blink paradigm, where two stimuli must be attended in rapid succession, showed that GABA concentrations in PPC were linked to an inhibition of attentional orienting from the first to the second target^51^. One interpretation of our own findings could thus be that inhibitory mechanisms in the parietal cortex play a role in the control of selective attention, which in turn is a part of volitional control. This interpretation would be consistent with previous findings using fMRI. For auditory streaming, the activity in the right intraparietal sulcus (mostly coincident with our PPC voxel) was correlated with attention and awareness of the competing streams, the two being confounded in this study^28^. In a visual paradigm, the activity of the intraparietal sulcus has been found to be modulated by instruction-guided attention, whereas the activity of the visual areas was positively correlated with the visibility of visual targets, regardless of attentional levels^52^. In EEG studies, activity in the right inferior parietal cortex was elicited before perceptual switches of the Necker cube^53^ and binocular rivalry^54^, suggesting a causal link. Hence, it is plausible that the parietal cortex contains subregions for manipulating perceptual content.

Interestingly, unlike previous suggestions^33,34^, we could not relate volitional control to the PFC function. Note that we manipulated volitional control with instructions to select one perceptual interpretation^35^, and not with instructions to speed up or slow down the alternations^32–34^. This was motivated by the hypothesis that instructions to speed up may be mediated by general attention or arousal effects, whereas percept selection is more specifically related to bistable competition^35^. When this distinction is made, it does not seem that PFC is implicated in the initiation of perceptual switches themselves^55^.

### Predictive coding

Current formulations of hierarchal perception usually appeal to some form of hierarchal predictive processing or predictive coding^56–59^. This perspective ties together several themes we have referred to above. In brief, in predictive coding, perception is cast as Bayesian belief updating through the use of prediction errors. In other words, expectations about the causes of sensory input are used to generate top-down predictions. These predictions are then compared with the actual input (or expectations at lower levels in cortical hierarchies) to form a prediction error. This prediction error is then returned to revise expectations - produce better predictions and eliminate prediction error. Crucially, the influence of prediction errors is controlled by optimising their precision (i.e., reliability or inverse variance). Technically, predictive coding can be regarded as a form of Kalman filtering and the precision corresponds to the Kalman gain applied to prediction errors. This later formulation is relevant to the present findings from several perspectives; first, it links cortical gain and the inhibition-excitation balance to the precision of ascending prediction errors in cortical hierarchies. Physiologically, this rests upon the post-synaptic gain and the sort of synaptic balance found in our spectroscopy measurements. Psychologically, the modulation of precision is generally thought of in terms of attentional gain or selection^60^, which provides a nice link between the inhibition-excitation balance and the attentional mediation of volitional control for perceptual organisation. Furthermore, it is simple to show that the precision corresponds to the rate of evidence accumulation in predictive coding, which fits neatly with our results pertaining to the duration of perceptual switches^61^. Multistable perception in the context of predictive coding has already been discussed^62^. When the precision of lower level prediction errors is attenuated in a modality-specific sensory cortex, the rate of evidence accumulation is suppressed and the duration of any given multistable percept increases. In contrast, during volitional control, the precision of higher level prediction errors is attenuated (in the parietal cortex), thereby rendering parietal expectations more sensitive to the sensory features of interest^63^. This interpretation is potentially important because many neuropsychiatric symptoms can now be understood in terms of aberrant precision control, particularly conditions such as autism and schizophrenia^64^. It may be no coincidence that a loss of inhibition-excitation balance is now considered one of the primary pathophysiologies of schizophrenia^65,66^.

### Integrated-distributed models of perceptual organisation

The account emerging from the current findings is of inhibitory-excitatory processes^13^ mediating neural competition within sensory regions, additionally modulated by higher-order mechanisms such as volitional control. This is highly consistent with long-standing and influential hierarchical models of perceptual organisation, where basic computational principles are implemented in a distributed manner^4^, with an additional modulation by higher-level processes such as attention or volition^67^. Using perceptual multistability and MRS imaging, we thus observed further experimental evidence for such models directly in human participants^68^.

## Materials and Methods

### Ethics statement

The study was carried out in accordance with the Declaration of Helsinki. All procedures reported in this study were approved by the Ethics and Safety Committees of NTT Communication Science Laboratories and ATR-Promotions (protocol nos: H24-004 and AN14-001). All participants gave written informed consent after the procedures had been fully explained to them.

### Participants

Thirty-six participants were recruited for the experiment (22 males and 14 females; *M*_age_ = 36.9, *SD*_age_ = 10.9). All participants were right-handed Japanese people with normal hearing and with normal or corrected-to-normal vision. None had any history of neurological or psychiatric disorders. All participants experienced an MRS session with AC, MT and PFC voxels. Due to the limited time available for an experimental session, another MRS session for the PPC voxel was run on a subsequent day, on a subset of 26 participants (20 males and 6 females, *M*_age_ = 34.1, *SD*_age_ = 8.3). Behavioural data were obtained immediately after the first MRS session.

### MRS data acquisition

To minimize confounding factors affecting the GABA and Glx concentrations, we conducted the acquisition of MR spectra at a fixed time for all participants, namely between 1:00 p.m. and 4:00 p.m. Data were acquired with a 3 T MRI scanner with a 12-channel receive-only head coil (MAGNETOM Trio, Siemens). Head motion was minimized by providing comfortable padding around the participant’s head. For an assessment of cortical thickness and volume, anatomical images were obtained with a T1-weighted pulse sequence (isotropic voxel size of 1 mm^3^).

MR spectra were acquired from four 3 × 3 × 3 cm^3^ voxels of interest, positioned in AC, MT, PPC, and PFC (Fig. 1). Voxels were positioned by using internal landmarks to achieve a consistent position between participants. The AC voxel was aligned with the first transverse sulcus. It contained the Heschl gyrus (Brodmann area: BA 41) and included the anterior part of the temporal plane (BA 42). The MT voxel (BA 19) was centred at the junction of the ascending limb of the inferior temporal sulcus and the lateral occipital sulcus. The PPC voxel was centred on the intraparietal sulcus (BA 7) of the right hemisphere. The voxel included a part of the superior and inferior parietal lobules. The PFC voxel was located at the anterior part of the middle frontal gyrus (BA 46). All the voxels except the PPC voxel were angled parallel to the surface of the left hemisphere.

Four runs (for each of the four regions of interest) were acquired for each participant from the different voxels. Before each run, we carefully carried out manual shimming (approximately 5 min) of the magnetic field in the voxel. We used the Mescher-Garwood proton resolved spectroscopy (MEGA-PRESS) technique^69^. For each spectrum, 64 spectral averages of 1024 data points were acquired with a repetition time of 1500 ms and an echo time of 68 ms, resulting in a scan duration of 3 min 18 sec (Supplementary Figure S1). An editing pulse with a bandwidth of 44 Hz was applied at 1.9 ppm (on) and 7.5 ppm (off) in interleaved scans. An unsuppressed water signal was also acquired from the same voxel. The difference in the edited spectra yielded the GABA and Glx peaks. As with all metabolic imaging studies using MRS, the GABA and Glx measures reflect both the intracellular fruid and the synaptic pool.

### Behavioural tasks

After the MRS data acquisition, the participants performed the multistability tasks outside the scanner (Fig. 2A). The behavioural experiment lasted approximately 1 hr. Stimulus presentation and response collection were managed using MATLAB with the Psychophysics Toolbox^70^. Two tasks were conducted separately: auditory streaming and moving plaids^23^.

The auditory stimuli were made from repetitions of an ABA- tone pattern, where A and B represent two different pure tones and the hyphen represents a silent interval. The A and B tones were centred on 1 kHz with a four-semitone frequency difference between them (frequency for A = 891 Hz; frequency for B = 1122 Hz). The duration of each tone was 40 ms, including 10-ms rising and falling cosine ramps. The stimulus onset asynchrony between successive tones was 100 ms. The presentation level was set at 70 dB SPL. Stimuli were delivered through Sennheiser HDA 200 headphones.

The visual stimuli consisted of two rectangular-wave gratings (velocity = 1.25 deg/sec; spatial frequency = 0.5 cycle/deg; duty cycle = 0.5). The gratings were moving in directions 120 deg apart. The stimuli were presented through a circular aperture on a grey background, at a viewing distance of 57 cm and covering a visual angle of 5 deg. A small point for fixation was added to the centre of the visual stimuli.

We first explained the two tasks using a visual illustration of the stimuli. For auditory streaming, participants were asked to report whether they heard one stream (ABA-ABA-…) with a galloping rhythm, or two streams (A-A-… and -B–B–…) with an isochronous rhythm for each stream. For plaids, participants were asked to report whether they saw a single plaid moving upward, or two superimposed gratings moving sideways in opposite directions. In the first part of the experiment, participants were simply instructed to pay attention to the stimulus (Neutral instructions). They reported their perception continuously during a 5-min presentation of each stimulus. Their responses were collected via two buttons on a computer keyboard. A response indicated by a button press was held until a subsequent button press.

Following the Neutral condition, two further conditions were run with volitional control manipulations^16^. In the Grouped condition, the participants were instructed to try as far as possible to maintain perception of one stream or one plaid. In the Split condition, the participants were instructed to try to maintain perception of two streams or two gratings. The order of the two volitional conditions was randomized across the participants.

### Data analyses

The MRS data were analysed using TARQUIN (version 4.2.10)^71^. The data were Fourier-transformed to a spectrum of 2048 data points, the signal was smoothed by a 3 Hz Lorentzian filter, phased and referenced to a water signal at 4.7 ppm. A basis set in the software was fitted to the average spectrum allowing peak amplitudes, widths, and frequencies to be optimised (Voigt function). The final results were expressed as GABA and Glx signals (peaks at 3.00 and 3.76 ppm, respectively) relative to the unsuppressed water signals. The GABA and Glx concentrations were quantified in arbitrary units (a.u). The GABA and Glx concentrations (mean ± standard error) were 1.78 ± 0.07 a.u. and 1.22 ± 0.04 a.u. for AC; 1.47 ± 0.05 a.u. and 1.31 ± 0.05 a.u. for MT; 1.44 ± 0.06 a.u. and 1.36 ± 0.08 a.u. for PPC; 1.53 ± 0.09 a.u. and 1.13 ± 0.05 a.u. for PFC. GABA/Glx ratios were computed to assess the inhibition-excitation balance in the voxels.

For the behavioural data analysis, time-series of percept durations were analysed separately for each auditory streaming and plaid motion block. All durations were longer than 300 ms. It has been previously reported that the duration of the first percept is longer than the duration of subsequent percepts for auditory streaming and moving plaids^16,72^. Thus, we excluded the duration of the first percept from the analysis. From the remaining durations, we evaluated the proportion of grouped percepts relative to the total duration. The raw distributions of the percept durations in all cases are provided as Supplementary Figure 2. Volitional control, as expected, changed the percept duration distribution. The amount of effective volitional control was estimated for each participant by subtracting the proportion of grouped percepts between blocks with grouped and split instructions^35^. Next, we computed the median of percept durations for each condition and participant. The pooled durations did not follow a normal distribution (Kolmogorov-Smirnov tests, *p* < 0.01), so we transformed the data to a logarithmic scale to enable us to perform a repeated-measures ANOVA. The Šidák correction was used for post hoc comparisons (*α*-level = 0.05). Pearson correlation coefficients and 95% CIs were computed between the behavioural and MRS measures. Statistical analyses were carried out with IBM SPSS Statistics (version 22) and R (version 3.1.2).

## Electronic supplementary material

Supplementary Information

## Data Availability

Data will be made available on request.
