# Supplementary material for: Inhibition-excitation balance in the parietal cortex modulates volitional control for auditory and visual multistability
Source: Sci Rep. 2018 Sep 28;8:14548. doi: 10.1038/s41598-018-32892-3 (PMC6162284; doi:10.1038/s41598-018-32892-3)
Supplement: Supplementary file 1 — Supplementary Information [file 41598_2018_32892_MOESM1_ESM.pdf]

Supplementary Information

Inhibition-excitation balance in the parietal cortex modulates volitional control for auditory and visual multistability

Hirohito M. Kondo[1,2], Daniel Pressnitzer[3,4], Yasuhiro Shimada[5], Takanori Kochiyama[5,6] & Makio Kashino[7,8]

[1]School of Psychology, Chukyo University, Nagoya, Aichi 466-8666, Japan

[2]Human Information Science Laboratory, NTT Communication Science Laboratories, NTT Corporation, Atsugi, Kanagawa 243-0198, Japan

[3]Laboratoire des Systèmes Perceptifs, CNRS UMR 8248, 75005 Paris, France

[4]Département d'Études Cognitive, École Normale Supérieure, 75005 Paris, France

[5]Brain Activity Imaging Center, ATR-Promotions, Seika-cho, Kyoto 619-0288, Japan

[6]Department of Cognitive Neuroscience, Advanced Telecommunications Research Institute International, Seika-cho, Kyoto 619-0228, Japan

[7]Sports Brain Science Project, NTT Communication Science Laboratories, NTT Corporation, Atsugi, Kanagawa 243-0198, Japan

[8]School of Engineering, Tokyo Institute of Technology, Yokohama, Kanagawa 226-8503, Japan

	GABA				Glx			
	AC	MT	PPC	PFC	AC	MT	PPC	PFC
Percept duration								
Auditory streaming	0.40*	0.05	-0.35	-0.17	-0.40*	-0.33	-0.25	-0.19
Moving plaids	-0.01	0.45**	-0.06	-0.08	0.16	-0.38*	-0.04	-0.10
Volitional control								
Auditory streaming	0.31	-0.20	0.08	-0.18	-0.03	-0.29	-0.35	0.05
Moving plaids	0.14	0.21	0.28	-0.16	-0.03	-0.20	-0.17	-0.00

Table S1. Correlations between behavioural measures and GABA- and Glx-alone concentrations. Abbreviations: AC; auditory cortex, GABA; γ-aminobutyric acid, Glx; glutamate-glutamine, MT; motion-sensitive area, PFC; prefrontal cortex, PPC; posterior parietal cortex. ** $p < 0.01$, * $p < 0.05$.

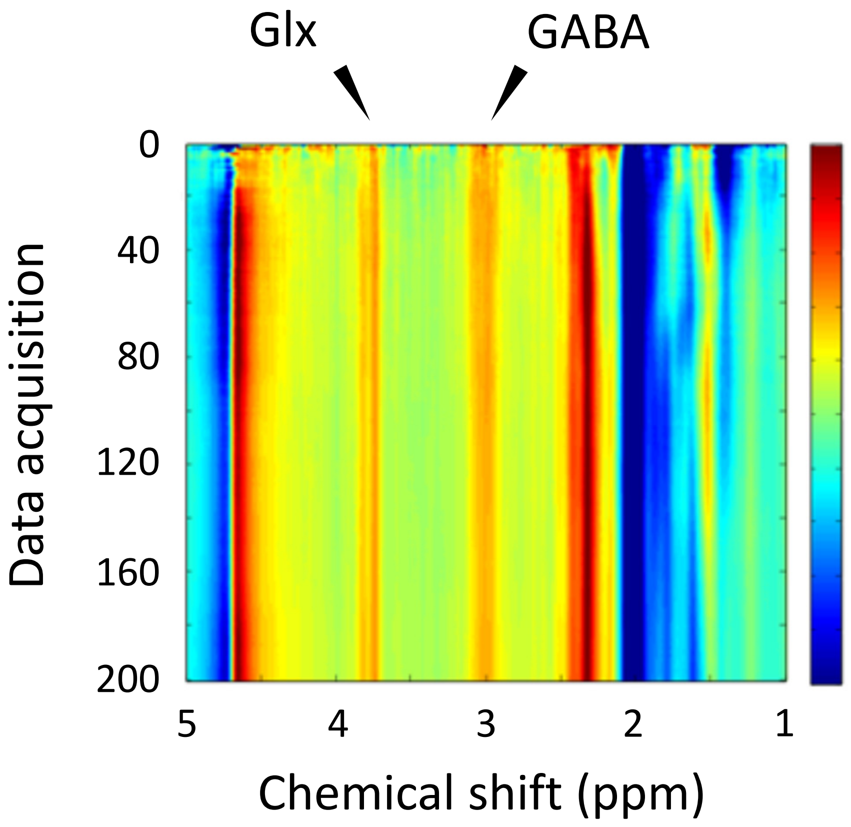

Figure S1. Temporal changes in MR spectra for a representative participant. In a preliminary study, we determined that the data quality of 64 spectral averages generally reached a satisfactory level. Data were obtained from the differences in spectra by editing radio frequency on/off pulses.

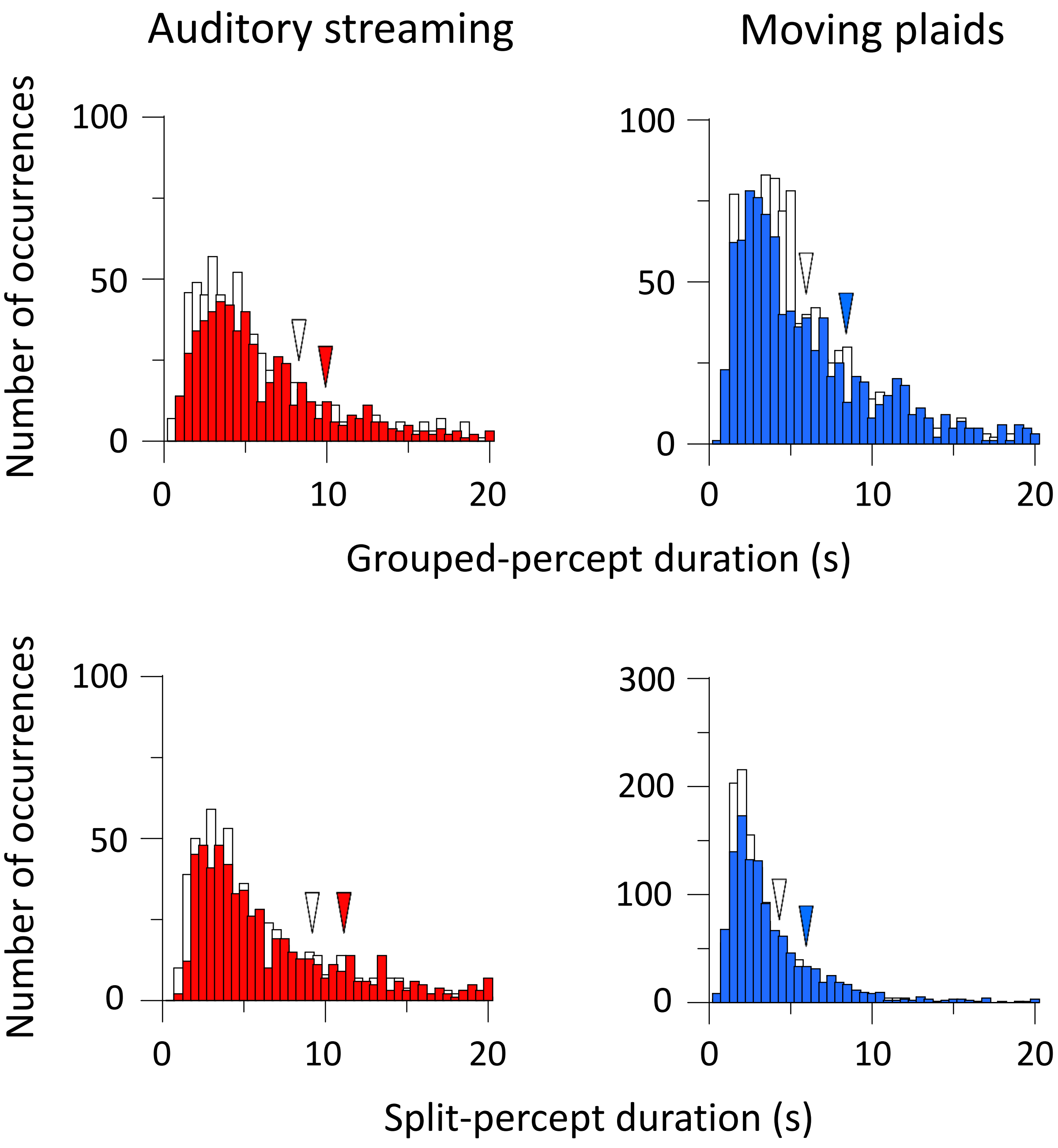

Figure S2. The histograms of percept durations. Coloured bars indicate percept durations under the Grouped and Split conditions, whereas open bars represent those under the Neutral condition. The data are compiled from all participants ($N = 36$). Triangles indicate the average of median percept durations for each participant.
